# Prevalence and Determinants of Road Traffic Accidents in Saudi Arabia: A Systematic Review

**DOI:** 10.7759/cureus.51205

**Published:** 2023-12-27

**Authors:** Eissa Z Alenezi, AlHanouv M AlQahtani, Sultan F Althunayan, Abdulrahman S Alanazi, Abdulrahman O Aldosari, Aqeel Mohammed Alharbi, Sulaiman T Alanazi, Salem Saad Sulaiman Alanazi, Hassan Ghazi Ali Tubayqi, Talal A Taheri

**Affiliations:** 1 General Practice, North Medical Tower Hospital, Arar, SAU; 2 Emergency Medicine, North Medical Tower Hospital, Arar, SAU; 3 Optometry, Arar Central Hospital, Arar, SAU; 4 Internal Medicine, North Medical Tower Hospital, Arar, SAU; 5 Dentistry, Vision College, Riyadh, SAU; 6 Dentistry, Prince Abdullah bin Abdulaziz bin Musaed Dental Center, Arar, SAU; 7 General Surgery, North Medical Tower Hospital, Arar, SAU; 8 Rehabilitation Medicine, North Medical Tower Hospital, Arar, SAU; 9 Epidemiology and Public Health, North Medical Tower Hospital, Arar, SAU

**Keywords:** seatbelt, determinants, prevalence, saudi arabia, traffic, road accidents

## Abstract

Globally, the frequency of road traffic accidents (RTAs) is sharply rising. It is concerning that the number of RTAs in the Kingdom of Saudi Arabia (KSA) has risen within the past ten years. As a result, laws governing things like speeding and seat belt use must be implemented to ensure driving safety. This study aims to determine the prevalence and determinants of road traffic accidents in Saudi Arabia. A thorough search was carried out in November 2023, mostly using PubMed, in compliance with PRISMA criteria. The search was limited to English-language research examining the causes of road traffic accidents and their prevalence. Certain inclusion and exclusion criteria were developed to guarantee the quality and applicability of the evaluated research. A wide spectrum of research from Saudi Arabia was included in the study without focusing on a specific gender. A discernible pattern indicated a high proportion of individuals affected by road traffic accidents. According to the findings of our investigation, there is growing evidence that Despite recent improvements in the incidence of road accidents, there is still significant variation in the incidence of accidents in Saudi Arabia. These results indicate that further study is needed to understand road accident prevention better.

## Introduction and background

Road traffic accidents (RTAs) have become a significant public health concern that requires quick attention. RTAs cause the bulk of hospital admissions due to trauma globally. The incidence of trauma caused by RTA was the highest cause of trauma-related admission (62.5%) [1]. These RTAs have the potential to cause harm to one or more bodily parts, and in extreme circumstances, they may even be fatal. They are a major contributor to early-life impairment and death. Saudi Arabia has the greatest RTA-associated mortality [2]. RTAs account for 13% of the Saudi population's disability-adjusted life years (DALYs) [3].

The World Health Organization (WHO) estimates that 1.25 million people die in RTAs each year, despite constant attempts to lessen the toll these incidents have had on healthcare systems. Twenty to fifty million individuals experience non-fatal injuries, such as trauma and disability, necessitating extended hospital admissions. By 2030, traffic accidents are predicted to rank seventh in terms of causes of mortality if ongoing efforts to enhance healthcare are not made [4].

In Australia, the UK, and the USA, the percentage of road traffic deaths is less than 1.7%; nevertheless, in the Kingdom of Saudi Arabia (KSA), they make up 4.7% of all fatalities [5]. In a similar vein, during the past ten years, the number of road deaths in KSA has climbed from 17.4 to 24 per 100,000 people. For males between the ages of 16 and 30 in Saudi Arabia, RTAs is thought to be the primary cause of mortality in the nation. With 19 fatalities and 4 injuries every hour in Saudi Arabia, automobile accidents pose a serious risk to public health [6].

Saudi Arabia is the biggest Arab state in Western Asia, with 2,149,690 km2. The Kingdom is a member of the "Group of Twenty" (G-20) of major economies and is classified as a high-income country. With a total population of over 27 million, of which one-fourth are foreigners, Jizan and Makkah have the greatest and lowest population densities (per km2), respectively, with 101 and 3.6 in Jizan and Al Jawf, respectively [7]. In Saudi Arabia, automobiles serve as the primary mode of transportation both inside and between cities. A recent estimate puts the number of automobiles on Saudi Arabia's roadways at over 6 million [8]. Eighty-one percent of hospital deaths in the Ministry of Health (MOH) are attributable to road traffic injuries (RTIs), whereas twenty percent of hospital beds are occupied by victims of RTAs. In RTAs, KSA has reported 86,000 fatalities and 611,000 injuries during the previous 20 years, with 7% of those injuries leading to lifelong impairments [9].

Depending on the kind of harm, RTIs can be classified into several categories. RTIs can be of any kind, ranging from cuts, piercings, and crush wounds to internal organ damage, fractures, and amputations. These injuries might impact almost any part of the body. The lower limbs were the most damaged body region in patients with RTAs, followed by the chest, upper limbs, head, and spine, per a study done between July 2014 and July 2017 [10]. According to different research, bigger, substantial organs, including the spleen, liver, and kidneys, are most frequently harmed by intra-abdominal lesions brought on by RTAs. There may be several benefits to this classification of RTI frequency based on the type of injury and the body location impacted. Estimating the death rate among RTA patients might be beneficial. According to published research, individuals with serious brain and pelvic injuries have a high death risk. As a result, the clinicians working in the emergency room could better assess the seriousness of each individual emergency case and properly prioritize them [11].

Previous research has shown that a number of factors can influence the frequency and severity of rear-end accidents (RTAs). These factors include the characteristics of drivers (such as age, gender, vision, and use of seat belts), the characteristics of vehicles (such as type, age, height, and tyre condition), the characteristics of the roadway (such as a number of lanes, lane width, shoulder width, pavement surface condition, and roadside condition), the characteristics of crashes (such as speed [12]. Several elements have been shown in previous research to provide effective countermeasures. Many preventative strategies have been successfully implemented worldwide to lessen the impact of RTAs [13]. Three main risks are linked to the high frequency and severity of rear-end collisions (RTAs): using a cell phone while driving, speeding, and not wearing seat belts [14]. According to data from earlier studies conducted in the Kingdom of Saudi Arabia, most of these collisions were caused by drivers exceeding the speed limit and breaking traffic laws, which may be avoided with tight enforcement [15].

The five road safety pillars established by the WHO are post-crash care, safe vehicle usage, road infrastructure, safe road safety management or policy, and safe vehicle usage. RTAs have largely been attributed to driver mistakes, poor vehicle conditions, and poor road conditions in various parts of Saudi Arabia. Due to a lack of localized, standardized data on RTAs, most of the injury-related death and disability measurements may be found in police reports, popular news stories, or WHO-predicted projections [16]. For example, Germany has reduced road fatalities by 69% and severity by more than 50% by developing and implementing appropriate preventative measures. Similarly, by enhancing the driving environment, reducing speed limits, and strictly enforcing traffic infractions, authorities in New York and several other American cities have been able to cut the number of deaths [17]. The Saudi Arabian government works nonstop to reduce the amount of people who die from traffic-related injuries. As part of the Vision 2030 initiative, the Saudi Ministry of Interior has set a goal to lower the number of road traffic fatalities to six per 100,000 people.

## Review

Methods

For this systematic review, the PRISMA (Preferable Reporting Items for Systematic Reviews and Meta-Analyses) standards were adhered to.

Study Plan and Length

November 2023 marked the completion of a systematic review.

Search Tactics

A thorough search was carried out across several databases, mainly using PubMed as the study search engine, to locate the pertinent studies. We limited our search to English results. To locate relevant studies, the following keywords were transformed into PubMed Mesh terms: "road traffic accidents," "prevalence," "women," "determinants," "Saudi Arabia," and "injuries ." Boolean logic's operations "OR" and "AND" were applied to match the necessary keywords. Human trials, articles written entirely in English, and publicly accessible materials were among the search results.

Selection standards: The inclusion criteria for this review included studies showing the prevalence of road traffic accidents and the determinants of these accidents, clinical studies, observational studies, easily accessible and free articles, and studies carried out in Saudi Arabia.

We excluded systematic reviews, meta-analyses, review articles, case reports, editor letters, and dispute resolution statements were not included. Studies outside Saudi Arabia and other languages besides English were also excluded.

Data extraction: Rayyan (QCRI) was used to identify duplicates in the search strategy output [18]. The role of Rayyan, a web-based software developed by Qatar Computing Research Institute (QCRI), in the review process is designed to assist researchers in conducting systematic reviews by facilitating the screening and data extraction process. 

The researchers filtered the combined search results using a set of inclusion/exclusion criteria to assess the relevancy of the titles and abstracts. Reviewers read all papers that satisfy the inclusion criterion in their entirety. The authors offered other methods for purposeful disagreement settlement. The research titles, authors, study year, nation, gender, participants, main outcomes, and conclusion were all gathered by the writers.

Technique for synthesizing data: A qualitative overview of the study's results and components was produced by creating summary tables using information from pertinent research. Following the data extraction for the systematic review, the most effective technique for using the data from the included study articles was selected.

Assessment of bias risk: A quality assessment of the included studies was conducted using the ROBINS-I risk of bias assessment approach for non-randomized treatment trials [19]. The following seven factors were assessed: confounding, selection of study subjects, classification of interventions, deviance from planned interventions, incomplete data, assessment of outcomes, and selection of the published result.

Results

**Figure 1:**
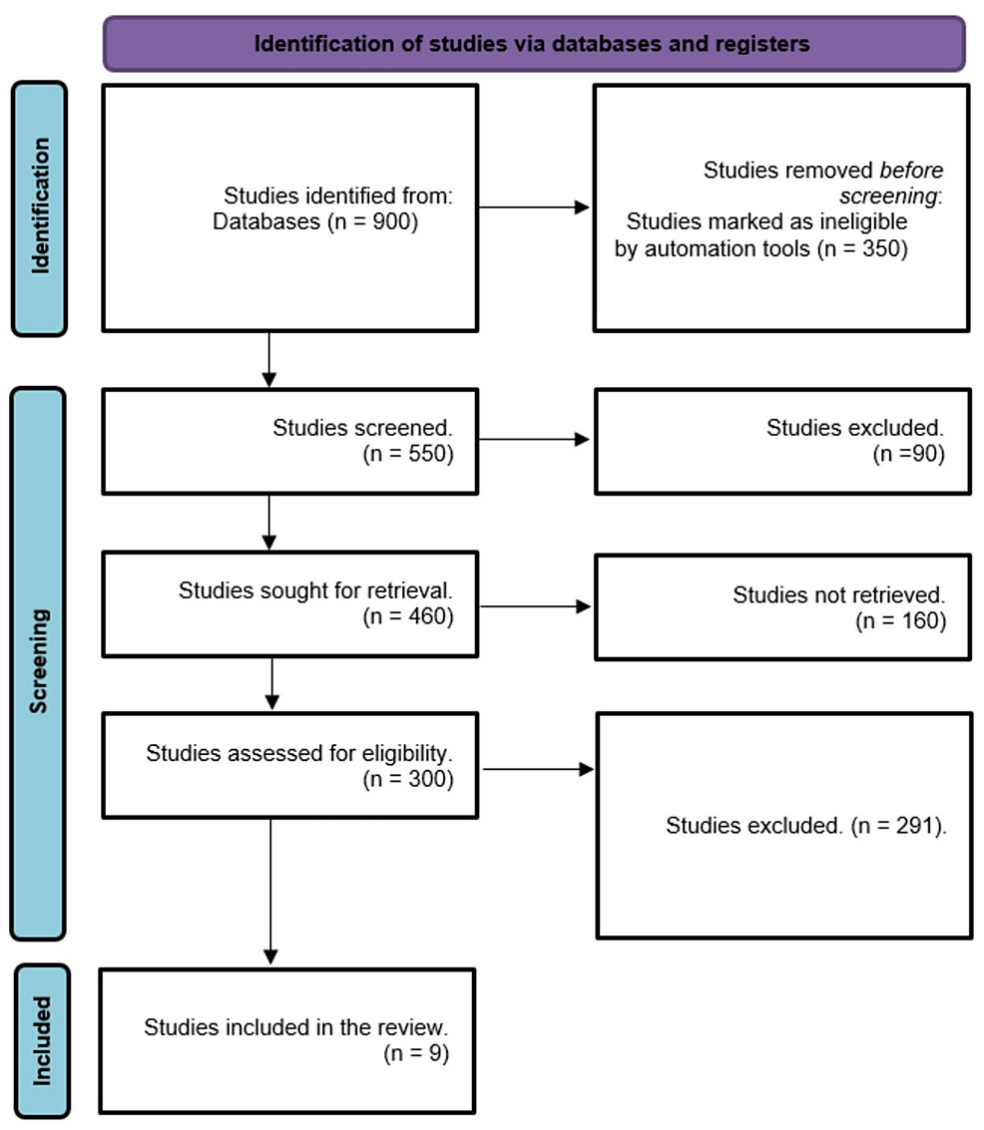
PRISMA flowchart summarizes the study selection process.

A total of 900 research articles were identified through the systematic search; 350 of those were automatically excluded. After screening the titles and abstracts of 550 papers, 90 studies were deemed unsuitable for publication. Only 460 publications were found out of the 160 studies that were requested for retrieval. 291 out of the 300 publications screened for full-text review were rejected due to inappropriate study designs or conclusions. Nine research papers met the eligibility criteria for this systematic review. A summary of the research selection process is depicted in Figure 1.

Characteristics of the Included Studies

Socio-demographic characteristics of participants: The socio-demographic information of participants from nine distinct studies is included in Table 1, which summarises the large number of cases from references [20-28]. These studies' geographic scope is constrained because they were all selected within Saudi Arabia's regions, like the Aseer region [22], Al Kharj [23], Riyadh [24, 26], Tabuk [25], Eastern Province [27], and Ha'il [28].

**Table 1:** Socio-demographic characteristics of the included participants.

Study	Area	Study design	No of cases	Age	Gender (Males)	Duration of study
Alslamah, Thamer et al. 2023 [20]	Saudi Arabia	Analysis of National Data	95,372	mean age = 30.13 (±12.86) years	59.1%	between 2016 and 2020
Alsofayan, Yousef Mohammad et al. 2023 [21]	Saudi Arabia	descriptive, retrospective cross-sectional observational study	160	NA	NA	January 2020 to July 2022
Algahtany, Mubarak Ali et al.2022 [22]	Aseer region	retrospective study	5797	Mean age of 30.7 years	87.28 %	1st January 2010 to 31 December 2019
AbdelRazik, Mohamed et al. 2021 [23]	Al‑Kharj	cross‑sectional study	300	NA	91%	between December 2018 and December 2019
Al Babtain, Ibrahim et al. 2022 [24]	Riyadh	Comparative Retrospective Study	688	Mean age =32.57 years	97.82%	March 2016 to March 2020
Yagoub, Umar et al. 2021 [25]	Tabuk	cross-sectional design	342	62.0% aged less than 36 years	100%	January 2018 to July 2018
Gorge, Jobby et al. 2020 [26]	Riyadh	cross-section study	1314	median age of 25	88%	2015 to 2017
Jamal, Arshad et al. 2019 [27]	Eastern Province	cross-sectional study	30,520	NA	NA	between 2009 and 2016
Ahmed S, Mahmood M, Rizvi SAH, et al. 2017 [28]	Ha'il	A retrospective study	10,855	NA	74.02%	January 1, 2016, to December 31, 2017

The research methodology of the reviewed studies [23,25-27] used a cross-sectional design; one study [20] was Analysis of national data, and the others [21,22,24,28] were retrospective studies.

The age range of participants had no distractions. The mean age recorded was 30.13 (±12.86) years, 30.7 years, and 32.57 years, according to Alslamah, Thamer et al. 2023 [20], Algahtany, Mubarak Ali 2022 [22], and Al Babtain, Ibrahim et al. 2022 [24], respectively. AbdelRazik, Mohamed et al. 2021 [23] reported that the age of 38% of cases was 20 to 30 years, and 23.3% were 30 to 40 years. Yagoub, Umar et al. (2021) [25] stated that 62.0% of cases were aged less than 36 years.

Saudi Arabian gender differences are evident in the country's driving regulations and habits. It should be mentioned that only men were permitted to use motor vehicles in Saudi Arabia for decades. Given that women have been able to drive since June 2019, it would be fascinating to look at the gender gap over time. As a result, all the studies concluded in this research had a higher percentage of men than women. Alslamah, Thamer et al. 2023 [20] study included 59.1% of the cases were men. Algahtany, Mubarak Ali. 2022 [22], AbdelRazik, Mohamed et al. 2021 [23], Al Babtain, Ibrahim et al. 2022 [24], Yagoub, Umar et al. 2021 [25], Gorge, Jobby et al. 2020 [26], and Ahmed S, Mahmood M, Rizvi SAH, et al. 2017 [28] studies were included: 87.28%, 91%, 97.82%, 100%, 88%, and 74.02%, respectively.

Accidental-related information and outcomes of the included participants: The aforementioned studies included the prevalence of RTAs in Saudi Arabia, the determinants of these accidents, and some outcomes (Table 2). Two studies by Alslamah, Thamer et al. 2023 [20] and Alsofayan, Yousef Mohammad et al. 2023 [21] explained in detail the prevalence of road traffic accidents among regions of Saudi Arabia. Riyadh was 25.3% and 44%, Mecca was 24.5% and 12%, and Medina was 6.4% and 7%, respectively. According to the study conducted by Jamal, Arshad et al. 2019 [27], Al-Ahsa had the highest average number of crashes per year (1,217 accidents). Of these, 612 occurred in Dammam, 432 in Hafr Al-Batin, 319 in Jubail, 257 in Dhahran City, and 80, 85, 121, and 237 in the cities of Ras Tanura, Khafji, and Al-Kobar. 

The determinants were observed in several studies. According to Alslamah, Thamer et al. 2023 [20], the collisions were 76%, roll-over caused around 15% of cases, and run-over caused 9%. Alsofayan, Yousef Mohammad et al. 2023 [21] stated that 90% of the accidents were due to direct collision, 3% were due to running a red light, and 1% were because of bad road conditions. Al Babtain, Ibrahim et al. 2022 [24] reported that 306 RTA were before the law on seat belts was implemented and 382 were after the implementation. Yagoub, Umar et al. (2021) [25] explained that 62.0% of participants had a vehicle-to-vehicle collision, and 32% of them reported that the use of a mobile phone was the main reason for their current road traffic collision. According to Jamal, Arshad et al. 2019 [27], abrupt lane changes accounted for 25.2% of collisions, over-speeding for 20.60%, driver distraction for 19.40%, vehicle overturning for 21.1%, and pedestrian strikes for 15.6% of incidents. According to Alslamah, Thamer et al. 2023 [20], age has an impact on the proportion of RTAs since RTAs are more common in younger persons. Additionally, Yousef Mohammad et al. 2023 [21] noted that in order to direct national preventative efforts and assist decision-makers in putting appropriate corrective steps into action, it is imperative that the relevant elements connected to the vector, driver behaviours, and the surrounding environment be taken into consideration. According to Algahtany, Mubarak Ali.2022 [22], there has been a consistent decrease in incidents with good consistency between police and health registration data as a result of speed restrictions and seat belt use. According to AbdelRazik, Mohamed et al. 2021 [23], RTAs are the main reason for admission to the ICU and account for 57% of all injury-related admissions, placing a heavy cost on the healthcare system. According to Al Babtain, Ibrahim et al. 2022 [24], the head and neck areas were the most often impacted body parts prior to seatbelt deployment, whereas the chest was the most frequently affected region following it. Road traffic accidents can result in serious injuries and fatalities, which have an adverse effect on the victims' and their families' emotional and physical well-being, social lives, and financial situation, according to Yagoub, Umar et al. 2021 [25]. According to Gorge, Jobby et al. 2020 [26], males were more likely than women to experience it. According to Jamal, Arshad et al. 2019 [27], a number of effective collision prevention and mitigation solutions have been put forth, including traffic enforcement, traffic calming measures, safety education initiatives, and key stakeholder cooperation. According to Ahmed S, Mahmood M, Rizvi SAH, et al. 2017 [28], the most frequent kind of injury was a fracture involving one or more sites.

**Table 2:** Accidental related information and outcomes of the included participants.

Study	Prevalence of accidents according to areas	Determinants	Other outcomes
Alslamah, Thamer et al. 2023 [20]	Riyadh 25.3%	collisions 76%	young people are more prone to RTAs
	Mecca 24.5%		
	western region 10.9%,	roll-over 15%	
	Aseer 10%		
	Medina 6.4%	run-over 9%	
	Qassim 5.2%		
Alsofayan, Yousef Mohammad et al. 2023 [21]	Riyadh 44%	90% direct collision	Understanding contributing factors related to the vector, driver behaviors, and the surrounding environment is crucial to guide national preventive measures and help decision-makers to implement proper corrective actions.
	Eastern Province 15%	3% running a red light	
	Makkah 12%	1% bad road conditions.	
	Madina 7%		
Algahtany, Mubarak Ali et al.2022 [22]	NA	NA	speed limits and wearing of seat belts have resulted in a concordant decline in incidence with good consistency between police and health registration data.
AbdelRazik, Mohamed et al. 2021 [23]	NA	NA	RTAs contribute to 57% of all injury‑related admissions and are the principal cause of admission to the ICU, posing a significant burden on healthcare
Al Babtain, Ibrahim et al. 2022 [24]	NA	306 had an RTA before the law on seat belts was implemented	the most common body region affected before the seatbelt implementation was the head and neck regions (43.13%), and the most common region affected after the implementation was the chest (47.37%)
		382 after the implementation	
Yagoub, Umar et al. 2021 [25]	NA	62.0% of participants had a vehicle-to-vehicle collision	Road traffic collisions can cause severe injuries and fatalities, which leave a negative impact on the mental and physical health, social life, and financial conditional of the victims and their families.
		32% of the participants reported that use of a mobile phone was the main reason for their current road traffic collision	
Gorge, Jobby et al. 2020 [26]	NA	NA	Men had a higher incidence than women (19% vs. 12%).
Jamal, Arshad et al. 2019 [27]	Al-Ahsa had the highest mean number of annual crashes (1217):	25.2% Sudden Lane changes	various suitable crash prevention and mitigation strategies, for example, traffic enforcement, traffic calming measures, safety education programs, and coordination of key stakeholders, have been proposed.
	612 in Dammam	20.60% over-speeding	
	432 in Hafr Al-Batin	19.40% driver distraction	
	319 in Jubail	21.1% vehicle overturning	
	257 in Dhahran city	15.6% hitting pedestrians	
	The cities of Ras Tanura 80, Khafji 85, Al-Naimiyah 121, Al-Kobar 237		
Ahmed S, Mahmood M, Rizvi SAH, et al. 2019 [28]	NA	NA	Fractures of one or more sites were the most common type of injury, encountered in 47.66% patients.

Discussion

In every nation on the planet, road accidents and the injuries they inflict are a major source of morbidity and mortality. Road traffic accidents are responsible for 4.7% of all deaths in the Kingdom of Saudi Arabia. In 2021, 87.5% of commercial motorbike riders in Southern Nigeria were engaged in traffic incidents. Prior to the research, 74.0% of Ghanaians reported being engaged in collisions within the previous year. The frequency of traffic accidents on Iranian roads was 51.50% by a systematic review, GIS, and meta-analysis research, and 54.7% of the study was done in Adama. The USA has an incidence of 10.6 per 100,000 population, while the UK has an incidence of 2.9 per 100,000 [29]. This may be explained by the rigorous observance of traffic laws and regulations and their proper application to guarantee both primary and secondary RTA prevention in these nations. In a similar vein, it has been claimed that Saudi Arabia has a greater accident-to-death ratio (32: 1) than other high-income states (283: 1) for RTA-related deaths [30]. With an accident-to-injury ratio of 8:6 compared to the global ratio of 8:1, road traffic accidents are said to cause the most severe injuries in this nation [31]. These figures show a clear difference between the KSA and other nations with comparable economies and resources in terms of the frequency of RTAs, their negative effects, and the injuries and fatalities that follow. This demonstrates how urgently the KSA's trauma treatment has to be improved. 

Notably, a systematic review of all data gathered over a two-decade period regarding road traffic accidents and admissions to hospitals with RTIs revealed a pattern wherein young males were found to be more affected than females in every study, with a relatively low ratio of 2:1 in more recent studies and an increased ratio of 4:1 in earlier studies [32]. There is a discrepancy in the incidence of RTAs between studies in Saudi Arabia and other countries. In Saudi Arabia, RTAs are prevalent in cities hosting large religious gatherings during the busiest month of Ramadan. This could be due to drivers becoming less patient due to increased traffic and hunger. In other places, accidents are concentrated between December and May, likely because drivers adhere to different schedules during exam months for schools and universities. Due to reliance on automobiles for mobility, various drivers who lack fundamental knowledge of local driving laws and safety procedures have emerged. The geographical nature of the country plays a major role. Younger ages are more impacted since young people in Saudi Arabia view driving as a kind of fun as there aren't many other options, or they choose to utilize them, such as gyms, sports stadiums, or amusement parks. In residential neighborhoods, young people who drive illegally for short distances at high speeds on narrow roads may be more likely to be involved in accidents. The youth's actions reflect their lack of formal driving instruction, subpar sports facilities, and their parents' lack of discipline. In different studies, cortisol biomarkers were examined and linked to adolescent driving risk, calling for the creation of tailored intervention strategies. These reasons may be potential reasons for differences in RTA rates between Saudi Arabia and other countries [32].

On the other hand, Researchers in Ethiopia conducted a study that emphasized the importance of road traffic accidents (RTAs) as a serious public health issue, highlighting the need for stakeholders and government officials to pay attention to it [33]. Bucsuhazy and colleagues conducted another study that emphasized the complexity of accident causation and the significance of examining factors such as human behavior and environmental circumstances that lead to accidents [34]. In their study of motorbike accidents in Ethiopia, Oltaye and co-authors found a significant frequency of RTA patients, especially in males between the ages of 20 and 29 [35]. They also discovered significant correlations between age, sex, speed, and the type of road. The combined findings of these studies underscore the critical need for comprehensive approaches to address the substantial public health impact of RTAs, which should include education, enforcement, and infrastructure upgrades. These studies are consistent with the results of our study in Saudi Arabia, which also highlighted the need for a comprehensive approach to mitigate the impact of RTAs. Additionally, they similarly revealed trends in terms of the age and gender of RTA patients, further underscoring the need for targeted interventions to address these high-risk groups. Overall, it is clear that RTAs are a significant public health issue that requires urgent attention and action from all stakeholders.

Another important factor in reducing highway deaths is following traffic laws. Nearly three-quarters of all recorded crashes were vehicle collisions (36.3%), vehicle flipping (21.1%), and hitting pedestrians (15.6%). These crash categories were the most common. These figures appear to be at variance with those of research conducted in neighboring Oman, which found that the percentages of accidents, vehicle overturns, and pedestrian hits were, respectively, 58%, 4.8%, and 3.0% [33].

Using data from the Royal Oman Police national database, a recent study looked at the underlying effects of age and gender on the severity of RTA outcomes and discovered that young guys were the group most at risk of RTA [11]. They also stated that minor injuries from RTAs were more common among female drivers [11]. Both results emphasize the need for early gender-sensitive treatments and preventative measures to minimize the severity of RTA-associated damage and consequent impairment, with a priority to males. The findings of the Oman research are consistent with the findings of the current investigation [33].

The main conclusion of the Alsofayan, Yousef Mohammad et al. 2023 [21] study is that ambulance crashes are more common in Riyadh, the country's capital, while the Eastern area is second. The two holiest towns in Islam, Makkah and Madinah, have likewise seen an unusual number of ambulance crashes [34]. Since the number of accidents is consistent with research from other nations, the high population density, urbanization, and increased traffic frequency of major cities may cause the accidents [35]. According to recent Saudi Arabian research that examined national statistics on EMS response times for RTAs, Riyadh, the country's capital, had the most percentage of RTAs, followed by the Makkah area [7].

It would be appropriate to advocate for the primary prevention of road traffic accident (RTA) hazards to change young people's driving habits, especially through mass media campaigns. Health-related messages can be posted in public places like schools and malls. A suitable countermeasure against drivers who participate in dangerous driving behaviors should be developed in order to successfully minimize the size of road traffic events. Alcohol intake tests, drug usage, wearing a seat belt, driving speed, driver health, and vehicle maintenance conditions should all be routinely monitored in order to lower the number of traffic accidents [32].

Additionally, initiatives can be strengthened by strictly enforcing legislation in residential areas and providing other forms of entertainment, such as sports facilities, amusement parks, or health and fitness centers. All hospitals should have a uniform surveillance system in place to ensure simultaneous secondary and tertiary prevention, which is the hospital portion of post-crash treatment. Communities must be trained in first-response care for pre-hospital care components to reduce the length and complexity of RTAs. Identifying accident sites and risk factors and implementing relevant treatments can also be facilitated by using reliable Geographic Information Systems (GIS) [5,32].

Efforts to address the issue of road traffic accidents should also include collaboration with law enforcement agencies to ensure proper enforcement of traffic laws and regulations. This can be achieved through regular monitoring and evaluation of road safety measures, as well as the implementation of road safety audits to identify areas of improvement. By taking a comprehensive and multi-faceted approach, we can work towards creating safer road environments for all members of our community. This will lead to a better understanding of the causes and mechanisms of traffic accidents in our area, allowing for the development of initiatives to reduce the overall problem [32].

## Conclusions

In conclusion, the results of this review article clearly highlighted an indiscriminate description and explanations for RTAs in KSA compared to other countries. The major documented causes of RTAs were broken traffic regulations and a general lack of understanding of pedestrian safety. To support the present data suggesting the urgent need for implementing preventive programs, such as early awareness and education of young drivers, this study emphasized the factors description and reasons for RTAs in KSA, although An increasing amount of data points to the need for a thorough knowledge of the patterns and behavioral traits connected to RTAs both domestically and globally. In order to lessen the load, traffic laws should be enforced more strictly. This will help develop a standardized data system for identifying the core factors and strategies to cope with the problem. Accordingly, road safety may be improved in the future by advocating relevant primary preventive strategies in the population. Additionally, promoting a culture of responsible driving and fostering a sense of mutual respect among road users can positively impact reducing RTAs. By addressing these various factors comprehensively, we can work towards creating a safer and more secure road environment for everyone.
